# The Ethics of Veganism

**DOI:** 10.7759/cureus.56214

**Published:** 2024-03-15

**Authors:** Aryan Jaiswal, Tripti Shrivastava

**Affiliations:** 1 Nutrition, Jawaharlal Nehru Medical College, Datta Meghe Institute of Higher Education and Research, Wardha, IND; 2 Physiology, Jawaharlal Nehru Medical College, Datta Meghe Institute of Higher Education and Research, Wardha, IND

**Keywords:** ethical consumerism, ethical veganism, animal welfare, plant-based diet, veganism

## Abstract

In this article, we discuss the ethics of veganism by looking at the moral implications of having a vegan diet or plant-based diet. Veganism is a way of diet that forbids or avoids consuming any animal products. It has drawn a lot of interest recently due to awareness or trends of vegan diet, human health, and ethical behavior benefits. The aim of the research is to look into the moral values that talk about veganism, such as animal welfare, environmental sustainability, and human welfare. In this study, we conduct a thorough assessment of ethical theories and actual evidence in an effort to know about the ethical reasons for veganism and its larger societal impacts. Through an analysis of existing literature and clinical studies, we discuss the various challenges, advantages, lifestyle modification, and nutritional concerns related to a vegan diet.

## Introduction and background

Veganism is a way of life and a diet that forgoes any animal products in both food intake and other areas of one's life. Vegans avoid eating meat, dairy, eggs, honey, and other products made from animals. To lessen their influence on animal exploitation and suffering, they also frequently steer clear of goods like leather, fur, silk, and cosmetics that have been tested on animals. Donald Watson, a co-founder of the Vegan Society, invented the term "vegan" in 1944 as an expansion of vegetarianism to include avoiding all animal products. Typically, ethical, environmental, and health considerations form the foundation of veganism. Ethical vegans oppose the commercialization and exploitation of animals for human purposes and believe in the inherent worth and rights of all sentient beings. Vegans who care about the environment support a plant-based diet to lessen the environmental impact of animal agriculture. Though it needs careful planning to guarantee enough nutritional intake, healthy vegans embrace a vegan diet for the possible health benefits, such as decreased risks of heart disease, some malignancies, and obesity [1].

The origins of veganism may be found in earlier civilizations where some people followed vegetarian diets for moral, religious, and health-related reasons. However, Donald Watson and his companions formally invented the term vegan in 1944 when they established the Vegan Society in the United Kingdom. The current vegan movement officially began at this point. The ethical and health advantages of avoiding all animal products, such as meat, dairy, eggs, and honey, were emphasized by early vegan proponents. With the help of several organizations and advocacy groups, veganism eventually spread outside of the United Kingdom. Celebrity endorsements, rising public awareness of animal rights, environmental issues, and the health advantages of plant-based diets have all contributed to the rise of veganism in recent years. Today's varied worldwide population has embraced veganism as a vital ethical choice that reflects a dedication to sustainability, compassion, and the welfare of all living things [2].

Veganism has gained popularity and spread outside of the United Kingdom over the years. Around the world, a large number of organizations and advocacy groups have developed to promote veganism for a variety of causes, including animal rights and environmental sustainability. In recent years, veganism has attracted more attention as a result of celebrity, sports, and public endorsements, which has improved awareness and plant-based diet acceptance. The movement's deep-seated ethical and philosophical roots are clear from its historical backdrop, which also shows how it has developed and expanded in response to shifting societal norms, environmental concerns, and health issues. Today, veganism is still a well-known ethical position supported by individuals all over the world who want to build a society that is kinder and more sustainable [2].

Veganism represents a conscious and ethical choice to live in a way that reflects values of compassion, respect for animals, sustainability, and social justice. It encourages individuals to critically examine their actions, consumer choices, and the impact they have on the lives of animals, the environment, and other humans. While people may have different motivations for adopting a vegan lifestyle, the ethical foundations of veganism emphasize the importance of empathy, compassion, and the acknowledgment of the rights and interests of all sentient beings [3].

Animal rights, which maintain that all sentient species have intrinsic worth and should be treated with respect and compassion, are frequently the foundation of veganism [3]. This viewpoint is supported by the knowledge that animals are able to experience several types of emotions, including pain and pleasure [4]. 

The notion of non-harm or non-violence is one of the key ethical justifications for becoming a vegan. The goal of veganism is to reduce and finally end the exploitation and cruelty of animals used for human sustenance [5]. This involves mass-producing and killing animals for food, confining and abusing animals in factory farms, and using animals for experimentation or entertainment [6].

Vegans contend that these actions violate animals' fundamental right to an uncomplicated existence. They assert that animals should not be imprisoned, exploited, or slaughtered for human consumption and have an intrinsic right to enjoy their lives in the wild. People who choose a vegan lifestyle are choosing to live in accordance with their moral principles and to speak out against the needless suffering that animals endure in the food business [7].

Concerns about the environment provide yet another ethical justification for vegetarianism [8]. A significant factor in deforestation, water pollution, and climate change is animal agriculture. The ecosystem is harmed by the enormous amounts of land, water, and resources needed to produce and feed livestock. Plant-based diets, on the other hand, use fewer resources and leave a smaller carbon impact [9].

## Review

Methodology

**Figure 1:**
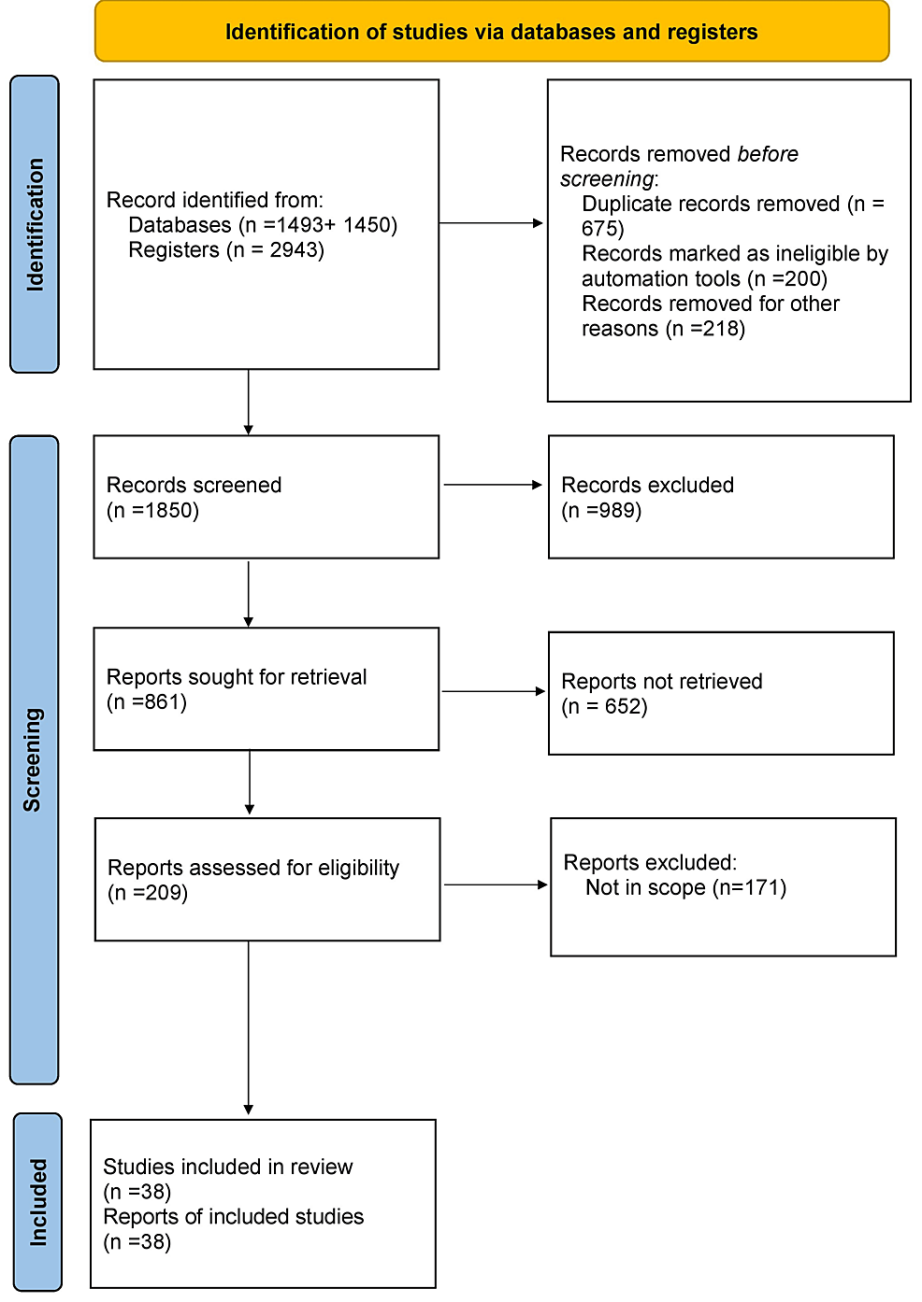
The selection process of articles used in this study Adopted from the Preferred Reporting Items for Systematic Reviews and Meta-Analyses (PRISMA)

The electronic databases PubMed and Google Scholar, in addition to Cureus and a few other open-access journal articles were used to conduct a search of the English language literature. We searched for and included as many relevant studies as possible using phrases like "vegan diet", "ethical veganism", "animal rights", "plant based lifestyle", "animal exploitation" and "impact of animal agriculture". The authors' expertise and experience in the subject area aided in archiving pertinent publications. This review article's goal is to discuss the benefits, challenges, impact on the lifestyle, and nutritional concerns of the vegan diet. A Preferred Reporting Items for Systematic Reviews and Meta-Analyses (PRISMA) flow diagram of the review indicating the screening process is summarized in Figure 1.

Health considerations of vegan diets

Health Benefits of a Balanced Vegan Diet

According to a 2019 review written by the American Heart Association and published in their journal, the Journal of the American Heart Association, one can lower their chance of developing coronary heart disease, diabetes, and numerous types of cancer by adopting a vegan diet. A study that appears in the Journal of Inner Medicine claimed that those who follow a vegetarian diet have a lower body weight and basal metabolic index (BMI) than people who follow a non-vegetarian diet. Plant-based diets with a high fiber content can help to support a balanced gut microbiota and enhance digestion.

Fruits, vegetables, and other plant-based foods strong in antioxidants, which can help guard against oxidative stress and inflammation, are often abundant in vegan diets. Plant-based diets may aid diabetics with better insulin sensitivity and blood sugar regulation. Eliminating animal products can also lead to lower cholesterol levels, reducing the risk of cardiovascular disease [8].

The vegan lifestyle and ethics of consumption

Animal Products in Non-food Industries

The vegan lifestyle extends beyond dietary choices to encompass various non-food industries that use animal-derived products. Vegans may avoid products like leather, fur, wool, silk, and other animal-based materials as these industries often involve the exploitation of animals and cause harm to them. Instead, they may opt for cruelty-free and eco-friendly alternatives, such as synthetic materials or plant-based fabrics. Additionally, some non-food products, such as cosmetics, household cleaners, and personal care items, may be tested on animals or contain animal-derived ingredients. Ethical vegans seek out products that are labeled as cruelty-free, vegan, and free from animal testing to align their consumption with their values [10].

Veganism and Consumer Choices

Vegans prioritize making ethical and sustainable consumer choices, considering not only the direct impact on animals but also environmental and social implications. They may opt for products with minimal environmental impact, support companies that align with their values, and avoid those associated with animal exploitation or harmful practices. In the context of food choices, vegans may prefer organic and locally sourced produce, as well as plant-based alternatives to animal products like plant-based milk, vegan cheeses, and meat substitutes. Vegans often navigate these ethical dilemmas by seeking the most compassionate and sustainable choices available to them, continuously educating themselves about the impacts of their consumption, and being open to continuous self-reflection and improvement in their ethical practices. The goal is to make choices that align with their values of compassion, respect for animals, and environmental consciousness while acknowledging the complexity of real-world trade-offs [10].

Ethical obligations of society and policy implications

Animal Welfare Laws and Regulations

It is ethically required of society to create and uphold rules and legislation that safeguard animal welfare. Animal welfare laws seek to stop the abuse, exploitation, and needless suffering of animals in a variety of contexts, including entertainment, labs, industrial farms, and pet ownership. These regulations may include issues including availability of food and water, humane treatment, and limitations on animal experiments. The consequences of the policy include bolstering current regulations governing animal welfare, enacting fresh legislation to address new problems, and ensuring efficient enforcement and compliance monitoring. Working together with stakeholders, specialists, and organizations dedicated to animal welfare can result in more thorough and solid regulations that take into account society's ethical concerns for animals [11].

The Role of Education and Awareness

Promoting humane treatment of animals and promoting a vegan lifestyle depends heavily on education and awareness. Society has an ethical duty to disseminate truthful information regarding diets that are plant-based, animal welfare, and the effects of animal agriculture on the environment. People may make wise and moral decisions about their dietary habits by encouraging empathy and understanding towards animals. The policy ramifications include incorporating environmental education and animal welfare into the school curriculum, sponsoring public awareness initiatives, and offering assistance to those considering adopting a vegan lifestyle. Government and non-governmental organizations can work together on educational projects to spread the word about moral concerns about the abuse of animals and the advantages of plant-based diets [11].

Encouraging Ethical Practices in Business

Businesses have a duty to follow moral guidelines that take into account the needs of people, the environment, and animals. The ethical duties of society include pressuring companies to be open about their labor practices, sourcing, and production procedures. By highlighting the value of sustainability and moral supply chains, businesses may be encouraged to give priority to ethical and sustainable practices. Implementing regulations that hold companies responsible for their environmental and social impacts, providing consumers with easy access to information about a company's ethical standards, and providing incentives and recognition to businesses that adopt ethical and sustainable practices are just a few of the policy implications. Additionally, governments should invest in sustainable and plant-based enterprises and encourage the study and creation of goods that don't use animal products [11].

We can all work together to create a more compassionate and sustainable world by acknowledging society's ethical responsibilities and putting ethical business practices, education, and animal welfare into practice. These initiatives promote a more moral and peaceful interaction between people and the natural environment, helping not just animals but also the earth and all of its inhabitants [11].

Criticisms and challenges to vegan ethics

Veganism may clash with cultural, religious, or social traditions that have strong ties to the consumption of animal products. Some communities may view veganism as impractical or incompatible with their cultural identity and dietary customs. Critics argue that shifting the entire global population to a vegan diet may not be feasible or sustainable in terms of food production and distribution. They raise concerns about the ability to meet the nutritional needs of a growing global population solely through plant-based diets. Critics question the extent to which individual consumer choices, like adopting a vegan diet, can bring about systemic change in industries and policies that drive animal exploitation and environmental degradation [12].

In summary, criticisms and challenges to vegan ethics stem from cultural, social, and systemic factors. To address these concerns, it is crucial for vegan advocates to consider cultural sensitivities, promote food security, and work toward systemic changes that align with ethical principles. A multifaceted approach that encompasses individual choices, policy advocacy, and sustainable solutions can contribute to building a more compassionate and sustainable world [12]. 

Addressing Common Misconceptions

A well-planned vegan diet may solve possible nutritional issues while providing a host of health benefits. For optimum health and well-being, vegans must be aware of their dietary choices, maintain sufficient nutrient intake, and seek advice from medical specialists or qualified dietitians. Common misconceptions regarding the vegan diet have been summarized in Table 1.

**Table 1:** Common misconceptions Source: [13]

Common Misconceptions	Description
Complete proteins	Contrary to popular belief, vegan diets do not lack complete proteins. When appropriately balanced, a range of plant-based meals may include all nine necessary amino acids
Nutritional deficits	Vegan diets can be nutritionally sufficient and satisfy all nutrient needs with careful planning and nutrient intake monitoring
Lack of energy	When a vegan diet includes a range of entire plant-based meals, it may be energy-dense and provide enough energy for everyday activities
Inadequate calcium	To promote bone health, vegans can get enough calcium via fortified plant-based milk substitutes, tofu, and dark leafy greens
Insufficient protein	When ingested in the right amounts and combinations, plant-based sources of protein may readily satisfy protein requirements

Discussion

Vegan diets can be a healthy and nourishing way to eat for people of all ages, including newborns, kids, and pregnant or nursing mothers. According to research, as long as they are carefully thought out and balanced, vegan diets can offer all the essential minerals like calcium, vitamin B12, vitamin D, iron, and protein [14-18].

Studies have shown that adopting a vegan diet can reduce the chances of risk of contracting high blood pressure, diabetes, coronary heart disease, and numerous types of carcinoma [19-23]. Fiber, antioxidants, and phytochemicals are often more abundant in vegan diets, which may aid in lessening inflammation and enhancing general health [24,25].

Vegans must, however, be mindful of their nutrient consumption and make sure they are getting enough of some nutrients, such as calcium, iron, and the B12 vitamin, which can be more challenging to obtain from sources that are plant-based [26]. Depending on their diet and lifestyle, some vegans might also need to supplement with omega-3 fatty acids or vitamin D [27]. Some of the nutritional concerns of the vegan diet have been summarized in Table 2.

**Table 2:** Nutritional concerns about vegan diets DHA: Docosahexaenoic acid; EPA: Eicosapentaenoic acid Source: [27]

Nutrients	Description
Protein	A range of protein-rich meals should be consumed by vegans to guarantee an appropriate intake of protein because plant-based sources of protein may be deficient in some critical amino acids
Vitamin B12	Vegans should think about taking B12 supplements or consuming fortified meals to prevent deficiency as B12 is largely found in animal sources
Omega-3 Fatty Acids	Plant-based omega-3 fat sources do not have enough DHA and EPA. Algal oil or pills can aid those who need omega-3
Iron	Heme iron from animal sources is more readily absorbed than non-heme iron from plant sources. Vitamin C and meals high in iron can improve absorption
Calcium	Although some plant-based diets include calcium, dairy products may have higher absorption rates. Calcium-rich foods and fortified substitutes should be consumed by vegans
Vitamin D	To preserve bone health, vegans who get little sun exposure may think about taking a vitamin D supplement
Zinc	It is crucial to eat foods high in zinc because plant-based sources of the mineral may have lesser bioavailability
Iodine	In order to achieve their iodine needs, vegans must use iodized salt or integrate foods high in iodine
Vitamin A	Beta-carotene, which is present in several fruits and vegetables, is a vegan source of vitamin A. When necessary, the body transforms beta-carotene into active vitamin A

Furthermore, vegan diets can still contain significant amounts of processed foods, sugar, and saturated fat, thus they are not necessarily healthful. It is crucial to minimize or avoid highly processed foods and added sugars while putting an emphasis on healthy, plant-based foods. In general, a well-designed vegan diet can offer all the nutrients required for optimum health and can have a number of possible health advantages [28]. Before making any significant dietary changes, one should speak with a healthcare provider or trained dietitian, especially if they have any dietary limitations or concerns [29].

An increasing corpus of studies indicates that a vegan diet can provide numerous health advantages over a non-vegetarian diet. Here are some vegan diet study results and strategies. Reducing the likelihood of chronic diseases: According to a 2019 review by the American Heart Association and published in their journal, the Journal of the American Heart Association, one can lower their chance of developing coronary heart disease, diabetes, and numerous types of cancer by adopting a vegan diet [30-34]. Lower BMI: A study that appeared in the Journal of Inner Medicine claimed that those who follow a vegetarian diet have a lower body weight and BMI than people who follow a non-vegetarian diet [35,36]. Increased nutrient intake: A larger intake of vegetables, fruits, grains, and seeds, which are all rich in minerals, vitamins, and fibers, is typical of vegan diets [37]. Reduced consumption of saturated fats: Animal products are the main source of saturated fats and have been related to an elevated risk of heart disease. Vegan diets often have a reduced consumption of saturated fats. Plant-based protein sources: Getting adequate protein on a vegan diet is a major issue, but there are several plant-based protein sources, including lentils, tofu, tempeh, and seitan [38].

Making the switch to a vegan diet requires careful meal planning to make sure one is getting adequate nutrition. Persons must think about working with a qualified dietitian who can advise on nutrient consumption and meal planning. Additionally, packaged vegan foods that might be heavy in sodium or sugar must be avoided. Limited intake of processed foods and an emphasis on whole foods is vital. Overall, compared to a non-vegan diet, a thoughtful vegan diet can offer a variety of health advantages, but it is crucial to watch out for processed vegan food and ensure one receives adequate nutrients [38].

## Conclusions

There are numerous justifications for choosing to follow a vegetarian or vegan diet. The most frequent causes are ethical considerations, environmental concerns, health advantages, animal compassion, religious or cultural precepts, etc. Many people refrain from using animal products because they feel it is unethical to slaughter or exploit animals for nourishment. Concerns about the environment include deforestation, water pollution, and greenhouse gas emissions caused by the meat industry. People can lessen their environmental impact by cutting out or limiting their meat consumption. When balanced and complete with all the required nutrients, a vegetarian or vegan diet can be healthful. Vegetarian and vegan diets have been demonstrated in studies to help lower the risk of heart disease, stroke, and several malignancies.

Some people merely adore animals and find it intolerable to think of them being harmed or murdered for food. Some religions or civilizations forbid the consumption of particular animals or animal products. Individuals must balance their ideas and values with their nutritional and health needs when deciding whether to consume just vegetarian or vegan food. 
